# Childhood vaccination coverage in Australia: an equity perspective

**DOI:** 10.1186/s12889-021-11345-z

**Published:** 2021-07-07

**Authors:** Arzu Arat, Hannah C. Moore, Sharon Goldfeld, Viveca Östberg, Vicky Sheppeard, Heather F. Gidding

**Affiliations:** 1 Institute of Environmental Medicine, Unit of Occupational Medicine, Karolinska Institute, Stockholm, Sweden; 2 Wesfarmers Centre of Vaccines and Infectious Diseases, Telethon Kids Institute, The University of Western Australia, Perth, WA Australia; 3 Department of Paediatrics, University of Melbourne, Melbourne, VIC Australia; 4 Department of Public Health Sciences, Stockholm University, Stockholm, Sweden; 5 Communicable Diseases Branch, Health Protection NSW, North Sydney, NSW Australia; 6 Women and Babies Research, Kolling Institute, Northern Sydney Local Health District, St Leonards, NSW Australia

**Keywords:** Vaccination coverage, Social inequities, Child health, MMR

## Abstract

**Background:**

This study describes trends in social inequities in first dose measles-mumps-rubella (MMR1) vaccination coverage in Western Australia (WA) and New South Wales (NSW). Using probabilistically-linked administrative data for 1.2 million children born between 2002 and 2011, we compared levels and trends in MMR1 vaccination coverage measured at age 24 months by maternal country of birth, Aboriginal status, maternal age at delivery, socio-economic status, and remoteness in two states.

**Results:**

Vaccination coverage was 3–4% points lower among children of mothers who gave birth before the age of 20 years, mothers born overseas, mothers with an Aboriginal background, and parents with a low socio-economic status compared to children that did not belong to these social groups. In both states, between 2007 and 2011 there was a decline of 2.1% points in MMR1 vaccination coverage for children whose mothers were born overseas. In 2011, WA had lower coverage among the Aboriginal population (89.5%) and children of young mothers (89.3%) compared to NSW (92.2 and 92.1% respectively).

**Conclusion:**

Despite overall high coverage of MMR1 vaccination, coverage inequalities increased especially for children of mothers born overseas. Strategic immunisation plans and policy interventions are important for equitable vaccination levels. Future policy should target children of mothers born overseas and Aboriginal children.

## Introduction

In Australia, childhood vaccination levels have increased substantially in the past few decades. Despite high overall vaccination coverage, pockets of under immunisation exist; with an increase in measles cases over the last 5 years [1]. In both the media and the scientific literature, vaccine hesitancy has been the main focus when tackling this issue [2–4]. However, vaccine hesitancy is only part of the explanation. Recent studies from Australia [5] and Europe [6] have shown social inequities to be more important predictors of vaccination coverage levels. In particular the Australian study [5] showed that the majority of incompletely vaccinated infants did not belong to a family rejecting vaccines, but to parents who were experiencing socioeconomic barriers to immunisation.

Pockets of under-immunised groups are a serious threat to controlling the spread of vaccine preventable diseases such as measles. Outbreaks of measles have been reported in the past decade in many countries among groups of non-immunised individuals from certain ethnic groups [7] and children of parents with certain religious beliefs [8, 9]. Measles among the under-immunised groups can put vulnerable children, such as infants that are too young for measles vaccination and individuals with immune deficiencies, at risk. Studying the social distribution of vaccination coverage is therefore crucial in identifying these pockets of under immunised groups and preventing future outbreaks.

As stated in the UN’s Sustainable Development Goals (SDGs), the issue of under immunisation is not only about differential impact of disease on vulnerable children, but also about the crucial goal of no child being left behind [10]. This requires identifying current social inequalities in vaccination coverage and assessing possible macro-level factors that could be playing a role in the observed patterns, such as the structure and organisation of healthcare services, vaccination policies, strategic immunisation plans, and the funding of vaccination programmes [11].

Even in countries with universal free vaccination programs, there is inequitable vaccination coverage [12]. In Australia, all childhood vaccinations that are part of the National Immunisation Program (NIP) are free-of-charge [13], including the first dose of measles vaccination since 1975 [14]. Despite this, between 2002 and 2013 (the period of interest to this study), several national reports have shown the Aboriginal population to have lower levels of measles-mumps-rubella (MMR) vaccination coverage when compared to the national average [15]. Furthermore, several studies have analysed other socio-economic factors and found them to be of importance for vaccination coverage [5, 16, 17]. However, there is still a gap in knowledge regarding the size and trends in social differences for MMR vaccination coverage with respect to other factors and whether there are regional differences.

The World Health Organization (WHO) recommends that countries seeking to eliminate measles should achieve at least 95% coverage of both doses of MMR [18]. The Australian NIP schedule recommends the first dose of MMR vaccination (MMR1) be received at 12 months of age [13]. This study assesses trends in MMR1 vaccination coverage according to various socio-demographic factors among children born between 2002 and 2011 in two Australian states: Western Australia (WA) and New South Wales (NSW). The socio-demographic context and composition, and to some degree the vaccination policies, healthcare structures, and strategic immunisation plans, differ between these two states which suggest a useful state level comparative approach. While previous studies typically focus on immediate factors such as parental attitudes and behaviours [19, 20], this study contributes to the literature by focusing on structural and organisational factors in relation to social inequities in vaccination coverage.

## Methods

This study used a register-based dataset in which the birth registrations and perinatal data records in WA and NSW were linked to the Australian Immunisation Register (AIR) using probability matching, with 99% linkage accuracy [21]. The choice of these two states was based on their established linkage capacity [22]. The details of the linkage process have been described previously [21]. This linkage enabled the analysis of additional socio-demographic factors that were unavailable on AIR.

### Study population

The study population was derived from all live births with both a birth registration and perinatal record in WA and NSW (97.5% of live births in the perinatal data collections) between 2002 and 2011 [21]*.*

Children receiving MMR1 by 2 years of age were considered vaccinated, as previously defined [12, 16, 23]. Children up to 2 years of age were studied in order to include also those who were vaccinated after the recommended age.

### Socio-demographic variables

The choice of socio-demographic variables was based on previous literature [5, 24] and available information in the linked national datasets. All of the socio-demographic variables were obtained from the perinatal data records. *Aboriginal and/or Torres Strait Islander *(herein respectfully referred to as Aboriginal) status was derived using a multi-stage median algorithm based on all linked data-sets except deaths, as described previously [21]. *Mother’s country of birth* was dichotomised as Australia vs non-Australia and *maternal age at birth* was categorised into five age groups (< 19, 20–24, 25–29, 30–34 and 35+ years). When presenting data graphically by year of birth, two maternal age groups were collapsed (25–29 and 30–34 years) to enhance clarity. *Socio-economic status* (SES) was measured by a relative area level deprivation scale, namely the Index of Relative Socio-Economic Advantage and Disadvantage (IRSAD). The IRSAD is a measurement at area level, composed of 17 variables that include information on income, education, unemployment and access to an internet connection [25]. This variable was presented in five categories, as done previously [17], with index scores ranging from below the 10th percentile (most disadvantaged) to above the 90th percentile (least disadvantaged). For graphical presentation, the variable was further collapsed into four categories by creating a single group composed of the 26th–90th percentiles. *Remoteness* was measured through the Accessibility/Remoteness Index of Australia (ARIA), defined by accessibility to services based on road distance and categorised as major cities, inner regional, outer regional, remote, and very remote area [26]. For graphical presentation, the remote and very remote areas were collapsed due to statistical power issues. Classification of both IRSAD and ARIA were based on the mother’s reported residential address at the time of birth.

### Statistical analysis

The percent of children vaccinated was determined based on the individual vaccination status of each child. We have calculated the percent coverage by dividing the number of children that received their MMR1 vaccination before 2 years of age by the number of births that were eligible for vaccination in that cohort, which is according to established methods [12, 16, 23]. Children who died before 2 years of age were excluded. The socio-demographic distribution of vaccination coverage was calculated for each state and birth year (2002–2011) with 95% confidence intervals (CIs) for proportions vaccinated provided for each category. However, as the CIs were very narrow and the proportions represent population coverage, comparisons were not based on statistical significance. All analyses were done in Stata and data were accessed through the Secure Unified Research Environment [27].

## Results

There were 1,973,203 children with a perinatal record in NSW and WA born between 1996 and 2012. Out of these 19,322 (0.98%) were removed because they lacked a corresponding record in the birth register, 17 were removed due to having their date of immunisation prior to date of birth and 5807 died before age of 2 years (total removed 1.3%). We then restricted the analysis to the most recent 10 year period with follow up to 2 years of age (births between 2002 and 2011). This restriction excluded 773,907 children and left 1,174,150 children in the study cohort.

For the 1,174,150 children born between 2002 and 2011, the average overall MMR1 vaccination coverage was 92.5% in WA and 93.2% in NSW and stayed relatively stable throughout the study period.

Table 1 presents vaccination coverage for the total study population in WA and NSW separately, stratified by socio-demographic factors. Children of mothers born overseas were found to have 3–4 percentage points lower vaccination coverage both in NSW and WA. Being unvaccinated was more common in Aboriginal children in both states, with a 3–6 percentage point difference, when compared to the non-Aboriginal population. In terms of maternal age, the lowest vaccination coverage was among children of mothers giving birth before age 20 years. In both states, vaccination coverage increased with increasing maternal age, apart from the oldest age-group which had a somewhat lower coverage than the preceding group. Similarly, vaccination coverage increased with decreasing socioeconomic disadvantage except for the least disadvantaged, which had lower coverage than the preceding group, although the differences were small (< 0.8%). For all socio-demographic indicators examined, the most disadvantaged groups had consistently lower coverage in WA than in NSW, except for children of mothers born overseas for whom the difference in coverage was small.

**Table 1:** Vaccination coverage by socio-demographic characteristic for children born between 2002 and 2011 in New South Wales and Western Australia

	New South Wales			Western Australia		
Characteristic	N (%Population)	%Vaccinated	CI (95%)	N (%Population)	%Vaccinated	CI (95%)
**Maternal country of birth**						
Overseas	266,527 (29.7)	90.7	90.6–90.8	81,448 (29.3)	90.1	89.9–90.3
Australia	626,848 (70.0)	94.5	94.5–94.6	195,032 (70.1)	93.5	93.4–93.6
Missing	2678 (0.3)	91.2	90.0–92.2	1617 (0.6)	92.6	91.4–94.0
**Aboriginal Status**^**a**^						
Yes	39,109 (4.4)	89.7	89.4–90.0	17,878 (6.4)	86.9	86.4–87.4
No	856,941 (95.6)	93.5	93.5–93.6	260,219 (93.6)	92.9	92.8–93.0
**Maternal Age (years)**						
< 20	31,798 (3.5)	90.4	90.1–90.8	13,480 (4.9)	88.8	88.3–89.4
20–24	122,087 (13.6)	92.4	92.2–92.5	42,905 (15.4)	91.7	91.5–92.0
25–29	245,062 (27.3)	93.7	93.6–93.8	76,630 (27.6)	92.9	92.7–93.1
30–34	298,425 (33.3)	94.0	93.9–94.1	88,708 (31.9)	93.3	93.1–93.5
35+	198,681 (22.2)	93.1	93.0–93.2	56,374 (20.3)	92.2	92.0–92.4
**Socio Economic Status**^**b**^						
0–10%	97,888 (10.9)	91.9	91.7–92.1	22,505 (8.1)	90.3	90.0–90.7
11–25%	135,441 (15.1)	93.2	93.0–93.3	41,523 (14.9)	91.3	91.0–91.6
26–75%	427,532 (47.7)	93.8	93.7–93.9	128,026 (46.0)	92.9	92.8–93.1
76–90%	137,637 (15.4)	94.0	93.9–94.1	41,237 (14.8)	93.6	93.4–93.9
91–100%	86,053 (9.6)	93.4	93.2–93.5	22,409 (8.1)	92.8	92.4–93.1
Missing	11,502 (1.3)	85.2	84.6–85.9	22,397 (8.1)	91.9	91.6–92.3
**Remoteness**						
Major cities	688,471 (76.8)	93.6	93.5–93.6	186,569 (67.2)	92.4	92.3–92.5
Inner regional	146,864 (16.4)	93.1	93.0–93.2	30,349 (10.9)	93.3	93.1–93.6
Outer regional	46,946 (5.2)	92.9	92.7–93.1	22,675 (8.2)	92.6	92.3–92.9
Remote	4278 (0.5)	94.0	93.3–94.7	11,366 (4.1)	92.8	92.3–93.2
Very remote	333 (0.04)	88.6	85.2–92.0	4741 (1.7)	91.3	90.5–92.1
Missing	9161 (1.0)	84.0	83.2–84.7	22,397 (8.1)	91.9	91.6–92.3
**Total**	896,053	93.4	93.3–93.4	278,097	92.5	92.4–92.6

^a^In NSW, Aboriginal status was unknown for 3 individuals (not shown in table); ^b^ State specific quintiles. 0–10% most disadvantaged, 91–100% least disadvantaged

**Fig. 1:**
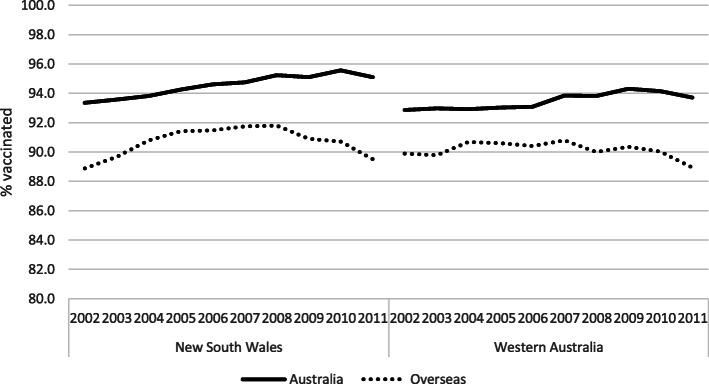
MMR1 coverage by year of birth and maternal country of birth, in New South Wales and Western Australia

Figure 1 presents the trend in vaccination coverage in both states, stratified by maternal country of birth. As seen in Table 1, children with a non-Australian born mother constituted a large part of the study population (29.6%). Over the entire period there were persisting inequities in vaccination coverage in both states which increased over time. For births in 2011, the coverage decreased to 89.3% among children whose mothers were born overseas in both states combined, leading to a difference of 5.4 percentage points from the children of mothers born in Australia.

**Fig. 2:**
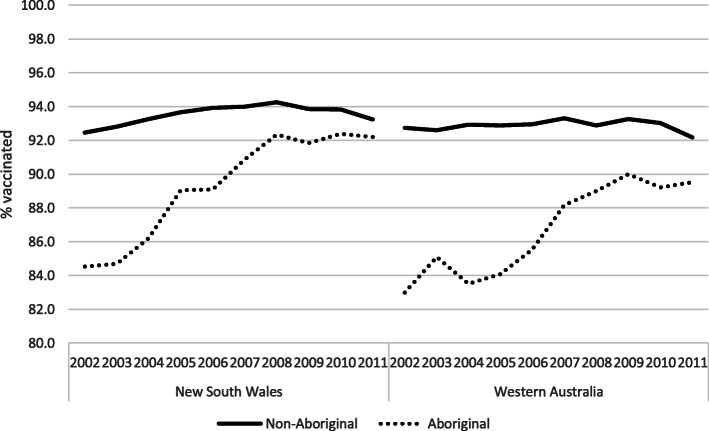
MMR1 coverage by year of birth and Aboriginal background, in New South Wales and Western Australia

Figure 2 shows the trend in MMR1 vaccination coverage stratified by Aboriginal status. At the beginning of the period, in both states the coverage in Aboriginal children was 84%; 8–10 percentage points lower compared to the non-Aboriginal population. Over time, there was an increase in coverage in Aboriginal children leading to a reduction in this gap in both states. The reduction was, however, greater in the case of NSW, when compared to WA; vaccination coverage for Aboriginal children born in 2011 in WA was 89.5% compared to 92.2% in NSW.

**Fig. 3:**
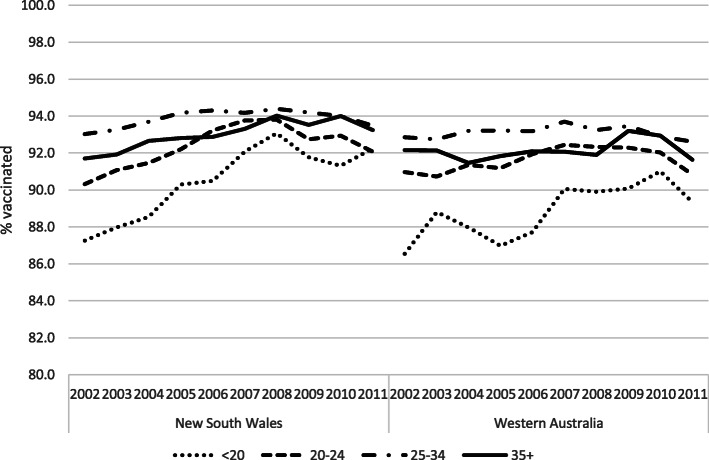
MMR1 coverage by year of birth and maternal age (in years) at birth, in New South Wales and Western Australia

The trend of MMR1 vaccination coverage by maternal age at birth is shown in Fig. 3. In NSW at the beginning of the period, children of mothers in the youngest age group had a 5 percentage points lower vaccination coverage compared to the rest of the study population. There was a steady reduction in this gap over the years, leading to approximately 93% coverage in all maternal age groups for children born in 2011. In WA, despite increasing coverage among children of mothers in youngest group, their levels were persistently lowest across the whole period. For births in 2011, coverage for WA children of the youngest mothers was 89.3%; 2.8 percentage points lower than other maternal age groups.

**Fig. 4:**
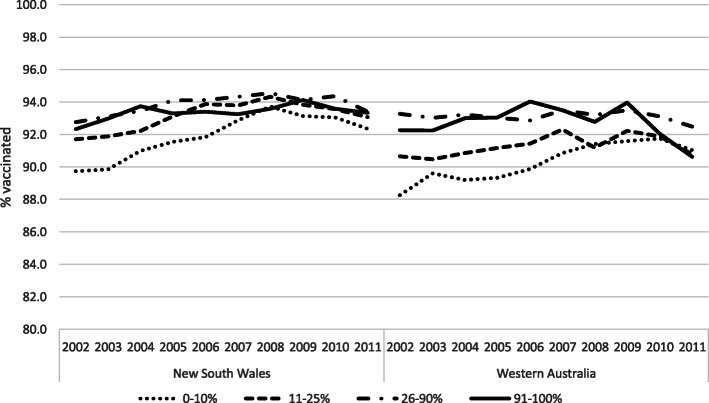
MMR1 coverage by year of birth and socioeconomic disadvantage, in New South Wales and Western Australia

Figure 4 presents vaccination coverage trends by level of socioeconomic disadvantage. Throughout the study period, inequities in NSW were smaller compared to WA. In NSW, starting with children born in 2008, the difference in coverage between the most disadvantaged group and the rest of the population was as low as 1 percentage point. In contrast to NSW, in WA the gap between the more disadvantaged socioeconomic groups (0–10% and 11–25%) and the rest of the population was around 3–4 percentage points and did not diminish until the later part of the period. There was a decline of 3.4 percentage points among the least disadvantaged children born after 2009 in WA, leading to 90.6% coverage: the same coverage as the most disadvantaged group.

**Fig. 5:**
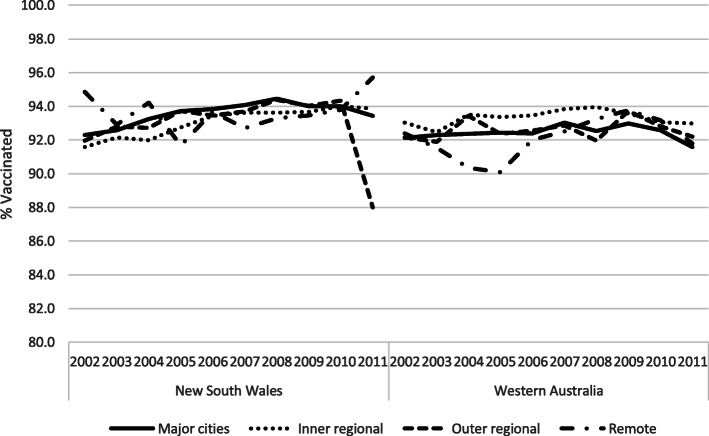
MMR1 coverage by year of birth and remoteness, in New South Wales and Western Australia

Figure 5 shows MMR1 vaccination coverage stratified by level of remoteness. Overall, remoteness did not seem to be an important predictor of inequalities in vaccination coverage. In NSW, no large differences were observed with respect to area of residence for births between 2002 and 2010, during which the coverage was approximately 92–94%. Among children born in 2011, there was a sharp decline in coverage for those residing in outer regional areas. In WA, there was a decline in vaccination coverage for births between 2002 and 2005 among the children living in remote areas. This geographical difference in coverage disappeared for children born in 2007 and onwards.

## Discussion

This register-based study was made possible by linkage of perinatal data and immunisation records of over 1.1 million children born in NSW and WA. It shows that despite an increase in vaccination coverage during the study period, the herd immunity level for the MMR vaccination (95%) was still not reached, especially in certain socio-demographic groups. Stratified analysis by maternal country of birth showed that, in both states, children of foreign-born mothers had persistently lower coverage of MMR1 than children of Australian-born mothers, with increasing inequalities since 2006. Stratified analysis of other socio-demographic indicators showed that over the years, the gaps in coverage have diminished, especially in NSW. In contrast, by the end of the study period certain inequalities remained among WA-born children, especially among the Aboriginal population and children of young mothers. For almost all birth years, coverage for most of the disadvantaged groups was lower for children born in WA when compared to NSW.

Children with foreign-born mothers constitute almost one third of our study population. Increasing the levels of vaccination in this group is therefore not only an issue of equity, but also an important opportunity to increase coverage at the population level. Our findings confirm the results of previous studies in Australia [16, 28, 29] and elsewhere [30]. The increasing differences in coverage within the study period may have multiple explanations. One possible factor is the change in the composition of the migrant population since 2006. There was increased migration from Southern Asian countries during the time period of analysis and a recent study of DTP3 coverage, using the same study dataset as our present study, showed declining on-time coverage since 2008 in children whose mothers migrated from South-East and Southern Asia for reasons that are unclear [31]. Another factor may be the healthcare system’s inability to adjust to the changing composition of the migrant population [32, 33].

Numerous studies have shown Aboriginal status to be another strong determinant of vaccination coverage [17, 34, 35]. However, in both states there was a considerable increase in vaccination coverage among Aboriginal children over the study period and a closing of the gap in coverage between non-Aboriginal and Aboriginal children. This was particularly evident in NSW where the implementation of local projects and state-level policy changes that focused on the Aboriginal population may have played an important role [36]. For example, since 2003 immunisation programs in NSW have sought to strategically integrate immunisation coordinators and Aboriginal health specialists [37], a process that did not begin in WA until 2015 [38]. The lower levels of coverage in Aboriginal children in WA might also be explained by the lack of a systematic state-level immunisation strategy in contrast to the two strategic plans developed in NSW [37, 39, 40]. Additionally, NSW implemented policy directives that required public health services to report to AIR and to follow up on children with overdue vaccinations. These directives may have played a role in increasing the vaccination coverage for the Aboriginal population to a greater extent in NSW and could serve as a model for tackling other social inequities.

Patterns of coverage by maternal age at childbirth mirror coverage disparities related to socioeconomic disadvantage. This is explained by the overrepresentation of disadvantaged backgrounds among the youngest mothers [41]. Furthermore, studies have shown Aboriginal mothers to be more likely to give birth at a younger age compared to mothers with a non-Aboriginal background [42, 43]. The lower vaccination coverage amongst the children with mothers in the oldest age-group compared to the preceding age group (Table 1) could be explained by older women having, on average, more children. A recent study by Gidding et al. [28] showed birth order to be one of the strongest predictors of delay in DTP vaccination with children with older siblings having the greatest delay.

An interesting finding was the decline in vaccination coverage in the least disadvantaged group in WA, observed during the last few years of the study period. Previous literature has shown that vaccine-hesitant parents often belong to advantaged socioeconomic groups, which might explain the decline observed in this study [44]. One reason for the absence of a decline in vaccination coverage in the least disadvantaged group in NSW could be due to how vaccine hesitancy is dealt with at an organisational level. For example, from the early 1990s, primary schools and day-care centres in NSW have required parents to provide documentation about the child’s immunisation status upon enrolment [45]. Additionally, educational programs within healthcare services [40] could also have helped to tackle vaccine hesitancy. Neither of these organizational changes were implemented in WA during this period.

Vaccination coverage by level of remoteness suggest that there is an equitable infrastructure for the provision of vaccines to remote areas in WA and NSW. One possible explanation for this could be the presence of local public health units and Aboriginal medical centres in remote regions. There are no obvious reasons that can explain the sharp decline in coverage for the last birth cohort living in outer regional areas of NSW. This observation needs further monitoring in more recent data to see if the pattern is continuing.

Our findings show that NSW has managed to reduce the social gap in coverage in relation to young mothers, Aboriginal background, and socio-economic disadvantage to a greater extent than WA. One reason for the disparity between the states might be because primary care doctors (GPs) have been the main vaccine providers in NSW since the mid-1990s, whereas in WA the delivery of vaccinations has been dispersed among multiple providers with the introduction of child and adolescent healthcare units in 2005 [14, 38]. Previous studies have shown that a unified and well-coordinated healthcare service can decrease social inequalities in vaccine coverage [46]. By minimising the need for parental knowledge and self-initiative, which are related to parents’ level of social disadvantage, such a healthcare service may make it easier for individuals who belong to a disadvantaged population to navigate the system and reach the services they need.

### Strengths and limitations

To the best of our knowledge, this is the first study to report trends in MMR1 vaccination coverage within social groups over a 10-year period across two states in Australia. This was made possible by linking the Australian Immunisation Register to individual level health data in which numerous social indicators were available for analysis [21].

Unlike most research on social inequalities in vaccination coverage, which studies complete immunisation levels for all recommended childhood vaccinations, our paper focuses specifically on the MMR vaccination. By analysing social inequalities in MMR vaccination coverage, we may be able to develop a more refined understanding of why measles cases have increased in recent years. However, the observed trends in MMR1 coverage are likely to apply to other childhood vaccines.

The study has limitations. The measurement of the socioeconomic status variable is at the area level (i.e. census collection district), which is composed of approx. Two hundred fifty households in an urban setting [47]. However, previous studies in Australia have shown this measurement to be at a small enough scale to draw valid conclusions [23].

The AIR is linked to Medicare enrolments (Australia’s universal public health scheme) which covers 99% of Australian residents by 12 months of age. At the time of this study, AIR recorded details of vaccinations (type, brand and date of administration) given to children < 7 years of age. As of 2016 AIR included all ages [48]. Due to possible underreporting (~ 2–3% in 2001), the levels of vaccination can be seen as minimal estimates of the actual coverage [49]. However, since then, completeness has reportedly improved in both states [50, 51].

The children who were excluded from the study population due to missing a birth registration record overrepresented Aboriginal children, children with younger mothers, mothers living in remote regions or with a low Socio-Economic Indexes for Areas (SEIFA) score [21]. However, since this group constituted only 1.3% of the total cohort population, we believe the study cohort is representative of all registered births and includes a large cohort involving 97.5% of all live births.

The aim of our study was to provide a descriptive analysis of trends over time in MMR1 coverage by 2 years of age by selected socio-demographic factors in 2 states with differing vaccination policies, healthcare structures, and strategic immunisation plans. Therefore, no multivariable analyses were conducted. We were unable to study some other relevant individual-level socioeconomic factors that could explain the observed results and it is possible that relative differences in coverage changed after 2 years of age due to catch up vaccinations. It would have been useful to have information on access to primary care and maternal vaccination coverage to further investigate the increasing inequities with respect to maternal country of birth, but these data were unavailable.

## Conclusion

This study points to an increase in coverage and generally a decrease in inequities in MMR1 vaccination during the study period. The exception was children whose mothers were born overseas, in whom coverage remained lower and inequities increased in 2006–2011, compared with children of Australian born mothers. This is valuable knowledge for designing programs and evaluating efforts to raise overall vaccination levels. Future research, using updated linkages of AIR and health datasets, is needed to determine the impact of more recent changes in legislation since 2016, specifically “No Jab No Pay” and “No Jab No Play” [52–54], on reducing inequities in vaccination coverage.

## Data Availability

The data that support the findings of this study was accessed through *Secure Unified Research Environment (SURE)* and restrictions apply to the availability of these data. Data were used under license for the current study, and so are not publicly available. The authors are, however, happy to share more detailed information about the dataset on request.

## Abbreviations

MMR: Measles-Mumps-Rubella
MMR1: First dose of Measles-Mumps-Rubella vaccination
NSW: New South Wales
WA: Western Australia
SES: Socio-economic status
IRSAD: Index of Relative Socio-Economic Advantage and Disadvantage
ARIA: Accessibility/Remoteness Index of Australia
SURE: Secure Unified Research Environment

## References

[1] Australian Government Department of Health (2020). Notifications-1991-Present, Australia (2.1).

[2] Grossman Z, Ashkenazi S, Rubin L (2017). How are we responding to vaccine-hesitant parents?. Lancet Child Adolesc Heal.

[3] Forbes TA, McMinn A, Crawford N, Leask J, Danchin M (2015). Vaccination uptake by vaccine-hesitant parents attending a specialist immunization clinic in Australia. Hum Vaccin Immunother.

[4] World Health Organization (WHO) (2018). Measles cases spike globally due to gaps in vaccination coverage.

[5] Pearce A, Marshall H, Bedford H, Lynch J (2015). Barriers to childhood immunisation: findings from the longitudinal study of Australian children. Vaccine..

[6] Danis K, Georgakopoulou T, Stavrou T, Laggas D, Panagiotopoulos T (2009). Socioeconomic factors play a more important role in childhood vaccination coverage than parental perceptions: a cross-sectional study in Greece. Vaccine..

[7] Mereckiene J, Cotter S, O’Flanagan D, Valentiner-Branth P, Muscat M, D’Ancona F (2012). Review of outbreaks and barriers to MMR vaccination coverage among hard-to-reach populations in Europe.

[8] Fournet N, Mollema L, Ruijs WL, Harmsen IA, Keck F, Durand JY, Cunha MP, Wamsiedel M, Reis R, French J, Smit EG, Kitching A, van Steenbergen JE (2018). Under-vaccinated groups in Europe and their beliefs, attitudes and reasons for non-vaccination; two systematic reviews. BMC Public Health.

[9] New York City Department of Health (2019). Measles-NYC Health.

[10] United Nations Department of Economic and Social Affairs. The 17 Goals-Sustainable Development. [cited 2020 Oct 11]. Available from: https://sustainabledevelopment.un.org/?menu=1300

[11] Wild C, Siciliani L, Barry M, Barros P, Brouwer W, De Maeseneer J (2018). Vaccination Programmes and health Systems in the European Union.

[12] Arat A, Burström B, Östberg V, Hjern A (2019). Social inequities in vaccination coverage among infants and pre-school children in Europe and Australia – a systematic review. BMC Public Health.

[13] Australian Government Department of Health (2020). National Immunisation Program Schedule.

[14] Australian Government Department of Health and Ageing (2010). Vaccine Preventable Diseases In Australia, 2005 To 2007.

[15] Australian Government Department of Health (2020). Immunisation coverage annual reports.

[16] Haynes K, Stone C (2004). Predictors of incomplete immunisation in Victorian children. Aust N Z J Public Health.

[17] Moore HC, Fathima P, Gidding HF, de Klerk N, Liu B, Sheppeard V, Effler PV, Snelling TL, McIntyre P, Blyth CC, ACIR Linkage Investigator Group (2018). Assessment of on-time vaccination coverage in population subgroups: a record linkage cohort study. Vaccine..

[18] World Health Organization (WHO) (2017). Measles vaccines: WHO position paper – April 2017.

[19] Crocker-Buque T, Edelstein M, Mounier-Jack S (2017). Interventions to reduce inequalities in vaccine uptake in children and adolescents aged <19 years: a systematic review. J Epidemiol Community Heal.

[20] Chow MYK, Danchin M, Willaby HW, Pemberton S, Leask J (2017). Parental attitudes, beliefs, behaviours and concerns towards childhood vaccinations in Australia: a national online survey. Aust Fam Physician.

[21] Gidding HF, McCallum L, Fathima P, Snelling TL, Liu B, de Klerk N (2017). Probabilistic linkage of national immunisation and state-based health records for a cohort of 1.9 million births to evaluate Australia’s childhood immunisation program. Int J Popul Data Sci.

[22] Moore HC, Guiver T, Woollacott A, de Klerk N, Gidding HF (2016). Establishing a process for conducting cross-jurisdictional record linkage in Australia. Aust N Z J Public Health.

[23] Hull BP, Mclntyre PB, Sayer GP (2001). Factors associated with low uptake of measles and pertussis vaccines — an ecologic study based on the Australian childhood immunisation register. Aust N Z J Public Health.

[24] Sato APS, Waldman EA, Tauil M de C (2016). Factors associated with incomplete or delayed vaccination across countries: a systematic review. Vaccine..

[25] Australian Bureau of Statistics (2018). Census of Population and Housing: Socio-Economic Indexes for Areas (SEIFA), Australia, 2016.

[26] Australian Bureau of Statistics (2018). Australian Statistical Geography Standard (ASGS): Volume 5 - Remoteness Structure, July 2016.

[27] SaxInstitute (2017). Introduction to SURE.

[28] Gidding HF, Flack LK, Sheridan S, Liu B, Fathima P, Sheppeard V (2020). Infant, maternal and demographic predictors of delayed vaccination: a population-based cohort study. Vaccine.

[29] Paxton GA, Rice J, Davie G, Carapetis JR, Skull SA (2011). East African immigrant children in Australia have poor immunisation coverage. J Paediatr Child Health.

[30] Wilson L, Rubens-Augustson T, Murphy M, Jardine C, Crowcroft N, Hui C, Wilson K (2018). Barriers to immunization among newcomers: a systematic review. Vaccine..

[31] Abdi I, Gidding H, Leong RN, Moore HC, Seale H, Menzies R (2021). Vaccine coverage in children born to migrant mothers in Australia: a population-based cohort study. Vaccine..

[32] Heywood A (2018). Improving vaccine coverage in hard-to-reach groups: immigrants and refugees. In University of New South Wales.

[33] Smith L (2015). The health outcomes of migrants: a literature review.

[34] Harris MF, Webster V, Jalaludin B, Jackson Pulver LR, Comino EJ (2014). Immunisation coverage among a birth cohort of Aboriginal infants in an urban community: immunisation paper. J Paediatr Child Health.

[35] O’Grady K-A, Krause V, Andrews R (2008). Immunisation coverage in Australian indigenous children: time to move the goal posts. Vaccine..

[36] Hendry AJ, Beard FH, Dey A, Meijer D, Campbell-Lloyd S, Clark KK, Hull BP, Sheppeard V (2018). Closing the vaccination coverage gap in New South Wales: the Aboriginal immunisation healthcare worker program. Med J Aust.

[37] New South Wales Department of Health (2003). NSW Immunisation Strategy 2003–2006.

[38] Government of Western Australia Department of Health (2013). Western Australian Immunisation Strategy 2013–2015.

[39] McIntyre PB, Durrheim DN, Campbell-Lloyd S (2010). The NSW immunisation strategy 2008-2011: how are we doing?. N S W Public Health Bull.

[40] New South Wales Department of Health (2008). NSW Immunisation Strategy 2008-2011.

[41] Australian Institute of Health and Welfare. Australia’s Children. Canberra; 2020. Available from: https://www.aihw.gov.au/getmedia/6af928d6-692e-4449-b915-cf2ca946982f/aihw-cws-69-print-report.pdf.aspx?inline=true

[42] Comino E, Comino E, Knight J, Knight J, Webster V, Webster V (2012). Risk and protective factors for pregnancy outcomes for urban Aboriginal and non-Aboriginal mothers and infants: the Gudaga cohort. Matern Child Health J.

[43] Australian Institute of Health and Welfare (2020). Australia’s mothers and babies data visualisations.

[44] Facciolà A, Visalli G, Orlando A, Bertuccio MP, Spataro P, Squeri R (2019). Vaccine hesitancy: An overview on parents’ opinions about vaccination and possible reasons of vaccine refusal. J Public Health Res.

[45] National Center for Immunisation Research Survaillance (2018). Significant events in immunisation policy and practice in Australia.

[46] Arat A (2020). Social inequalities in access to child healthcare services : an international comparative perspective.

[47] Pink B (2006). Information paper. An Introduction to Socio-Economic Indexes for Areas (SEIFA).

[48] Hull BP, Deeks SL, McIntyre PB (2009). The Australian childhood immunisation register-a model for universal immunisation registers?. Vaccine..

[49] Hull BP, Lawrence GL, MacIntyre CR, McIntyre PB (2003). Immunisation coverage in Australia corrected for under-reporting to the Australian childhood immunisation register. Aust N Z J Public Health.

[50] Law C, McGuire R, Ferson MJ, Reid S, Gately C, Stephenson J, Campbell-Lloyd S, Gabriel S, Housen T, Sheppeard V, Corben P, Durrheim DN, NSW Public Health Network AIR Study Group (2019). Children overdue for immunisation: a question of coverage or reporting? An audit of the Australian immunisation register. Aust N Z J Public Health.

[51] Australian Government (2017). National partnership on essential vaccines.

[52] Hull BP, Beard FH, Hendry AJ, Dey A, Macartney K (2020). “No jab, no pay”: catch-up vaccination activity during its first two years. Med J Aust.

[53] Australian Government Department of Health (2017). No Jab, No Pay – New Immunisation Requirements for Family Assistance Payments.

[54] National Center for Immunisation Research Survaillance (2020). No Jab No Play, No Jab No Pay.

